# Producers and Important Dietary Sources of Ochratoxin A and Citrinin

**DOI:** 10.3390/toxins5091574

**Published:** 2013-09-17

**Authors:** Vladimir Ostry, Frantisek Malir, Jiri Ruprich

**Affiliations:** 1 Center for Health, Nutrition and Food, National Institute of Public Health in Prague, Palackeho 3a, 612 42 Brno, Czech Republic; E-Mail: ruprich@chpr.szu.cz; 2 Department of Biology, Faculty of Science, University of Hradec Kralove, Rokitanského 62, 500 03 Hradec Kralove, Czech Republic; E-Mail: malir.frantisek@seznam.cz

**Keywords:** ochratoxin A, citrinin, producers, microfungi, dietary sources, foods

## Abstract

Ochratoxin A (OTA) is a very important mycotoxin, and its research is focused right now on the new findings of OTA, like being a complete carcinogen, information about OTA producers and new exposure sources of OTA. Citrinin (CIT) is another important mycotoxin, too, and its research turns towards nephrotoxicity. Both additive and synergistic effects have been described in combination with OTA. OTA is produced in foodstuffs by *Aspergillus* Section *Circumdati* (*Aspergillus ochraceus*, *A*. *westerdijkiae*, *A*. *steynii*) and *Aspergillus* Section *Nigri* (*Aspergillus carbonarius*, *A*. *foetidus*, *A*. *lacticoffeatus*, *A*. *niger*, *A*. *sclerotioniger*, *A*. *tubingensis*), mostly in subtropical and tropical areas. OTA is produced in foodstuffs by *Penicillium verrucosum* and *P*. *nordicum*, notably in temperate and colder zones. CIT is produced in foodstuffs by *Monascus* species (*Monascus purpureus*, *M*. *ruber*) and *Penicillium* species (*Penicillium citrinum*, *P*. *expansum*, *P*. *radicicola*, *P*. *verrucosum*). OTA was frequently found in foodstuffs of both plant origin (e.g., cereal products, coffee, vegetable, liquorice, raisins, wine) and animal origin (e.g., pork/poultry). CIT was also found in foodstuffs of vegetable origin (e.g., cereals, pomaceous fruits, black olive, roasted nuts, spices), food supplements based on rice fermented with red microfungi *Monascus purpureus* and in foodstuffs of animal origin (e.g., cheese).

## 1. Introduction

Ochratoxin A (OTA) is a very important mycotoxin. OTA is a nephrotoxic, hepatotoxic, embryotoxic, teratogenic, neurotoxic, immunotoxic, genotoxic and carcinogenic mycotoxin [1,2]. 

OTA exposure may lead to the formation of DNA adducts, resulting in genotoxicity and carcinogenicity (human carcinogens of the 2B group). Now, it seems that OTA could be “a complete carcinogen” (not only an initiator, but also a promoter) and that its mutagenicity has been revised, obliging reinforcement of its monitorization in food [3,4,5,6]. 

Recent OTA research is focused now on the new findings of OTA, like being a complete carcinogen, information about OTA producers and new exposure sources of OTA [2]. In the Czech Republic, one of the EU Member States, a new assessment of dietary exposure and health risk characterization of OTA is currently taking place for 10 population groups of both sexes aged 4–90 years (research project No. NT 12051–3/2011, entitled “Ochratoxin A—health risk assessment for selected population groups in the Czech Republic”) [2]. 

Citrinin (CIT), often found in the same food as OTA [7], is a powerful nephrotoxin. In repeat dose toxicity studies, the kidney was identified as the principal target organ for CIT, and significant species differences in the susceptibility to CIT have been observed [8,9]. The renal system of humans was found to be affected, and the mitochondrial respiratory chain was identified as a possible sensitive target for CIT [10]. A few studies have also addressed its potential for immunotoxicity [11]. Nevertheless, the studies of the immunotoxicity of CIT are rather incomplete, often non-specific and do not allow a conclusive evaluation. *In vitro* and *in vivo* studies provided clear evidence for reproductive toxicity and the teratogenic and embryotoxic effects of CIT [12,13,14,15,16,17,18,19,20,21,22]. CIT is not mutagenic in conventional bacterial assays, either with or without metabolic activation by the S9 fraction from rat or human liver or rat kidney [23]. CIT is not carcinogenic according to recent knowledge. The International Agency for Research on Cancer (IARC) (1986) concluded that there was limited evidence for the carcinogenicity of CIT to experimental animals and that no evaluation could be made of the carcinogenicity of CIT to humans. CIT is classified in group 3 (not classifiable as to its carcinogenicity to humans) [24], but has been shown to increase OTA carcinogenicity [25,26]. 

Recent CIT research is oriented toward nephrotoxicity; both additive and synergistic effects have been described in combination with OTA [2,27,28,29,30]. With regard to the nephrotoxicity of CIT, the situation can be complicated by the fact that CIT interacts simultaneously with other naturally occurring mycotoxins—e.g., OTA. Besides, CIT and OTA have also been associated with alterations in renal function and/or with the development of renal pathologies. It has been demonstrated that the co-exposure to CIT and OTA simultaneously modifies DNA adduct formation with increasing formation of the C-C8dG-OTA adduct [29]. The recent CIT research has focused on the instability of CIT during food processing. The low levels of CIT in processed foods may result from the fact that CIT is heat-sensitive and decomposes during heat treatment to form other complex compounds, such as CIT H1 and CIT H2, whose cytotoxicity, compared to the original CIT, is higher and lower, respectively [31,32,33].

## 2. Ochratoxin A and Citrinin Producers

### 2.1. OTA Producers

OTA is produced worldwide in foodstuffs by microfungi of the genera, *Aspergillus*, mainly in subtropical and tropical areas, and *Penicillium*, especially in temperate and colder zones [34,35,36,37,38,39,40,41]. These toxigenic microfungi almost always produce several toxins at the same time, for example OTA, OTB (ochratoxin B) or OTC (ochratoxin C) [42], and this simultaneous occurrence can result in synergetic toxic effects.

Due to considerable revisions in taxonomy, particularly within the genus *Penicillium*, and difficulties in correct species assignation to isolates within that genus, this identity has changed over time [43]. Table 1 and Table 2 give an overview of the current identity of microfungi *Aspergillus* and *Penicillium* species that are apparently able to produce OTA in foodstuffs [43,44].

**Table 1:** *Aspergillus* species as ochratoxin A (OTA) producers in foodstuffs.

Genera	Section	Species	Foodstuffs (example)
*Aspergillus*	*Circumdati*	*A*. *ochraceus* G. Wilh.	Soya bean, nuts, red pepper, cereals, green coffee beans
		*A*. *steynii* Frisvad & Samson	Coffee beans
		*A*. *westerdijkiae* Frisvad & Samson	Coffee beans
	*Nigri*	*A*. *carbonarius* (Bainier) Thom	Grapes, red pepper, coffee beans
		*A*. *foetidus* Thom & Raper	Grapes
		*A*. *lacticoffeatus* Frisvad & Samson	Coffee beans
		*A*. *niger* Tiegh	Grapes, peanuts
		*A*. *sclerotioniger* Frisvad & Samson	Coffee beans
		*A*. *tubingensis* Mosseray	Grapes

**Table 2:** *Penicillium* species as OTA producers in foodstuffs.

Genera	Subgenus	Series	Species	Foodstuffs (example)
*Penicillium*	*Penicillium*	*Verrucosa*	*P*. *verrucosum* Dierckx	Cereals
		*Verrucosa*	*P*. *nordicum* Dragoni & Cantoni	Dry ham, salami

Three major OTA producing species, *Aspergillus ochraceus*, *A*. *carbonarius* and *Penicillium verrucosum*, have quite different ecologies and physiologies, making it relatively easy to determine which species are responsible for OTA formation in a particular food or geographical location. 

In brief, *Aspergillus ochraceus* and closely related species grow at low water activities and at moderate temperatures. They are mostly associated with dried and stored foods, especially cereals. Although many papers describe *A*. *ochraceus* as the main producer of OTA, production by this and related species has not often been reported, and their importance appears to have been overstated [45]. 

The second producing *Aspergillus* species, *A*. *carbonarius* (and the closely related *A*. *niger*, which produces OTA less often), grows well at high temperatures and produces pigmented hyphae and spores, making it resistant to UV light. Consequently, *A*. *carbonarius* is commonly found in grapes and similar fruit that mature in sunlight and at high temperatures [45]. Black *Aspergilli* are considered as the primary source of OTA on grapes, produced on the berries during the growing season, mainly from maturing to ripening. In particular, *A*. *carbonarius* is the most important producer of OTA; however, *A*. *niger* and *A*. *tubingensis* can contribute to some extent in the vineyard. OTA contents can reach high levels in wine in some parts of the Mediterranean basin and in dried vine fruits in South America, Australia and Europe. OTA production is influenced by various factors, including climatic conditions/geographic areas, grape varieties/crop systems and berry damage caused by insects, fungal infection or excessive irrigation/rainfall. Fungicidal and insecticidal treatments can reduce infection by OTA-producing fungi and, consequently, OTA contamination [37,39,46].

The main food habitat for *Penicillium verrucosum* appears to be cereals grown in the cool temperate zones, ranging across Northern and Central Europe and Canada. It seems certain that growth of *P*. *verrucosum* in cereals is the major source of OTA in Northern Europe and other cool temperate zone areas.Higher amounts of OTA were produced on wheat than on other substrates, including maize, peanuts, rapeseeds and soybeans. *P*. *verrucosum* also produces CIT [45].

OTA can also be produced by toxigenic microfungi that grow on products made of pork meat during their ripening (*direct contamination*). *Penicillium nordicum*, a potent OTA producer, has been proven to grow on meat and meat products [47,48]. OTA is also found in meat products originating from animals that are fed with feedstuffs made from contaminated cereals as a major dietary component (*indirect contamination*) [49]. 

### 2.2. CIT Producers

CIT is produced worldwide in foodstuffs by microfungi of the genera, *Penicillium* [43,44,50] and *Monascus* [51]. Table 3 and Table 4 give an overview of the current identity of microfungi *Penicillium* and *Monascus* species that are apparently able to produce CIT in foodstuffs [43,44,50,51,52,53].

**Table 3:** *Penicillium* species as citrinin (CIT) producers in foodstuffs.

Genera	Subgenus	Series	Species	Foodstuffs (example)
*Penicillium*	*Furcatum*	*-*	*P*. *citrinum* Thom	Cereals, nuts, fruit
	*Penicillium*	*Expansa*	*P*. *expansum* Link	Fruit, cereals
	*Penicillium*	*Corymbifera*	*P*. *radicicola* Overy & Frisvad	Bulbs and root vegetables
	*Penicillium*	*Verrucosa*	*P*. *verrucosum* Dierckx	Cereals

**Table 4:** *Monascus* species as CIT producers in foodstuffs.

Genera	Species	Foodstuffs (example)
*Monascus*	*M*. *purpureus* Went	Food supplements with fermented red rice
	*M*. *ruber* Tiegh	Soya bean, sorghum, rice, oat

*Penicillium citrinum* is one of the commonest microfungi on Earth, occurring in all kinds of food and feed, in almost all climates. CIT is produced over the range of 15–30 °C and optimally at 30 °C. *Penicillium expansum* is known as a postharvest pathogen of fruits (e.g., apple) and vegetables. *P*. *expansum* also produces patulin [45]. 

A recent concern, although not related to CIT as a *Penicillium* toxin, is the presence of CIT in food colorings traditionally made in Asia from rice fermented with *Monascus purpureus* (“*red mold rice*”), which have been used for centuries for meat preservation and food coloring [51].

## 3. Important Dietary Sources of Ochratoxin A (OTA) and Citrinin (CIT)

### 3.1. Important Dietary Sources of OTA

The consumption of foodstuffs contaminated by OTA represents a major source of exposure to OTA in humans [54], while dermal contact or inhalation exposures to OTA show minor importance for the general population [55]. 

As such, OTA has been detected in foodstuffs of both plant and animal origin. In foodstuffs of plant origin, OTA has been found, in particular, in cereal products, beer, coffee, cacao, chocolate, spices (e.g., dried red pepper, chili powder, black pepper, cayenne pepper, nutmeg, coriander, ginger, curcuma), vegetables, green tea, pistachios, figs, raisins, grape juice, wine [56,57,58,59,60,61,62,63,64,65,66], liquorice and chestnuts [67,68]. Foodstuffs of animal origin, such as raw pork meat, pork blood products, kidney or poultry liver, are indirectly contaminated by OTA when animals are fed with contaminated feedstuffs [49,54,66,69]. However, meat products, such as raw ham muscle, cured meats, salami or dry-cured ham, may also be contaminated by OTA in a direct way. In particular, OTA is produced by the ochratoxigenic microfungi, *Penicillium nordicum*, growing on products made of pork meat during their ripening [47,48,54,69,70]. Cheese is also directly contaminated by OTA. The occurrence of OTA on the surface of hard cheese wheels has been repeatedly described in the literature [71]. The occurrence of OTA in blue cheese has also been reported. The available data clearly demonstrate that the contamination did not derive from contaminated milk, but it resulted from the molded spots of the ochratoxigenic microfungi (e.g., *Penicillium nordicum* as a contaminant in protein-rich food) on blue cheese [72].

The preliminary recent results of Czech research project No. NT 12051–3/2011 concerning the occurrence of OTA in foodstuffs of plant and animal origin in the years 2011–2013 are shown in Table 5 and Table 6 [66].

The occurrence of OTA in animal products is not generally considered to be of major public health concern. In line with this opinion, we do consider, preliminarily, the risk associated with the consumption of food derived from animals fed with OTA-contaminated feeds to be negligible.

The analytical results will serve as a basis for an assessment of dietary exposure and health risk characterization of OTA for ten population groups 4–90 years of age for both sexes. 

The dietary exposure of humans to OTA can be assessed by analyzing levels of OTA in biological materials, too. The use of biological markers in approximately the last two decades has indicated that humans are chronically exposed to OTA [3,73,74,75].

**Table 5:** The occurrence and amount of OTA in foodstuffs of plant origin.

Foodstuffs	*n*	*n*+%	Mean ^a^ (μg/kg)	Median ^a^ (μg/kg)	Range minimum-maximum (μg/kg)
hot red pepper	12	100	19.00	12.10	0.2–91.8
sweet red pepper	12	100	16.00	13.50	0.2–38.4
chili	12	92	6.70	3.43	0.1–32.7
spices mix	12	83	1.64	1.06	0.1–9.4
coffee instant	12	92	1.04	0.79	0.1–4.91
cocoa powder	12	50	0.94	0.31	0.1–4.1
black pepper	12	92	0.83	0.66	0.1–2.82
non-chocolate sweets	12	83	0.67	0.78	0.1–1.78
biscuits	12	58	0.57	0.22	0.1–1.69
raisins	12	42	0.46	0.10	0.1–2.17
rice	12	8	0.41	0.10	0.1–3.76
sponge biscuits	12	58	0.41	0.15	0.1–2.14
coffee	12	58	0.41	0.22	0.1–1.04
chocolate sweets	12	50	0.29	0.17	0.1–1.16
bitter chocolate	12	42	0.29	0.10	0.1–1.01
chocolate wafers	12	75	0.24	0.22	0.1–0.56
muesli	12	17	0.23	0.10	0.1–1.44
beer 10°	12	83	0.066	0.05	0.005–0.26
lager beer	12	100	0.064	0.05	0.01–0.18
red wine	12	25	0.069	0.005	0.005–0.7
white wine	12	42	0.017	0.005	0.005–0.036

Abbreviations: *n*+ (%), percentage of positive samples; ^a^ OTA levels < 0.2 μg/kg considered ½ limit of quantification (LOQ) = 0.1 μg/kg and OTA levels < 0.01 μg/kg considered ½ LOQ = 0.005 μg/kg, respectively (for drinks).

**Table 6:** The occurrence and amount of OTA in foodstuffs of animal origin.

Foodstuffs	*n*	*n*+%	Mean ^a^ (μg/kg)	Median ^a^ (μg/kg)	Range minimum-maximum (μg/kg)
pork kidney	12	8	0.13	0.10	0.10–0.46
pork meat	12	8	0.11	0.10	0.10–0.20
chicken liver	12	8	0.12	0.10	0.10–0.28

Abbreviations: *n*+ (%), percentage of positive samples; ^a^ OTA levels < 0.2 μg/kg considered ½ LOQ = 0.1 μg/kg.

### 3.2. Important Dietary Sources of CIT

CIT was found in foodstuffs of vegetable origin, e.g., cereals and cereal products, rice, pomaceous fruits (e.g., apples), fruit juices, black olive, roasted nuts (almonds, peanuts, hazelnuts, pistachio nuts), sunflower seeds, spices (e.g., turmeric, coriander, fennel, black pepper, cardamom and cumin) and food supplements based on rice fermented with red microfungi *Monascus purpureus* [51,59,76,77,78,79,80,81,82]. Cheese is also contaminated by CIT where toxigenic strains directly grow in the cheese mass [83]. An overview of previously reported literature data on the occurrence of CIT in foodstuffs in the years 1972–2010 was prepared by European Food Safety Authority (EFSA) [84].

The results of selected recent studies (2009–2013) on the occurrence of CIT in red mold rice are shown in Table 7.

**Table 7:** The occurrence and amount of CIT in red mold rice.

Foodstuffs	*n*	*n*+%	Mean (mg/kg)	Range minimum-maximum (mg/kg)	References
Red mold rice	1	100	-	1.43	[85]
Red mold rice	1	100	-	15.21	[86]
Red mold rice	12	33	-	24–189	[87]
Red mold rice	50	100	4.03	0.23–20.65	[88]

Abbreviations: *n*+ (%), percentage of positive samples.

In addition to CIT, several bioactive compounds (monascin, ankaflavin, lactone and acid forms of monacolin K) were determined to be in red mold rice used as an ingredient in food supplements [89]. The maximum level of the CIT in food supplements based on red mold rice is being prepared by the European Commission (Directorate General for Health and Consumers) right now [90].

## 4. Conclusions

All the recent information on both the “relevant” OTA and CIT producers and the new sources of exposure to OTA and CIT is very important for health risk assessment. It is recommended to promote the correct use of agrotechnological practices with regard to raw materials (e.g., good agricultural practices (GAP)) and processed products (hazard analysis and critical control points (HACCP)) in order to reduce the concentration of OTA and CIT in foodstuffs and to avoid the harmful effects resulting from the consumption of foods contaminated by OTA and CIT.

## References

[1] Pfohl-Leszkowicz A., Manderville R.A. (2007). Ochratoxin A: An overview on toxicity and carcinogenicity in animals and humans. Mol. Nutr. Food Res..

[2] Malir F., Ostry V., Novotna E. (2013). Toxicity of the mycotoxin ochratoxin A (OTA) in the light of recent data. Toxin Rev..

[3] Malir F., Ostry V., Pfohl-Leszkowicz A., Roubal T. (2012). Ochratoxin A exposure biomarkers in the Czech Republic and comparison with foreign countries. Biomarkers.

[4] Pfohl-Leszkowicz A., Manderville R.A. (2012). An update on direct genotoxicity as a molecular mechanism of ochratoxin A carcinogenicity. Chem. Res. Toxicol..

[5] Hibi D., Suzuki Y., Ishii Y., Jin M., Watanabe M., Sugita-Konishi Y., Yanai T., Nohmi T., Nishikawa A., Umemura T. (2011). Site-specific *in vivo* mutagenicity in the kidney of gpt delta rats given a carcinogenic dose of ochratoxin A. Toxicol. Sci..

[6] Akman S.A., Adams M., Case D., Park G., Manderville R.A. (2012). Mutagenicity of ochratoxin A and its hydroquinone metabolite in the *supF* gene of the mutation reporter plasmid pS189. Toxins.

[7] Pfohl-Leszkowicz A., Tozlovanu M., Manderville R., Peraica M., Castegnaro M., Stefanovic V. (2007). New molecular and field evidences for the implication of mycotoxins but not aristolochic acidin Human Nephropathy and Urinary tract tumor. Mol. Nutr. Food Res..

[8] Arai M., Hibino T. (1983). Tumorigenicity of citrinin in male F344 rats. Cancer Lett..

[9] Sándor G., Busch A., Watzke H., Reek J., Ványi A. (1991). Subacute toxicity testing of ochratoxin-A and citrinin in swine. Acta Vet. Hung..

[10] Ammar H., Michailis G., Lisovsky T. (2000). A screen of yeast respiratory mutants for sensitivity against the mycotoxin citrinin identifies the vascular ATPase as an essential factor for the toxicity mechanism. Curr. Genet..

[11] Sharma R.P. (1993). Imunotoxicity of mycotoxins. J. Dairy Sci..

[12] Hood R.D., Hayes A.W., Scammell J.G. (1976). Effects of prenatal administration of citrinin and viriditoxin to mice. Food Cosmetics Toxicol..

[13] Singh N.D., Sharma A.K., Dwivedi P., Patil R.D., Kumar M. (2007). Citrinin and endosulfan induced maternal toxicity in pregnant Wistar rats: Pathomorphological study. J. Appl. Toxicol..

[14] Singh N.D., Sharma A.K., Dwivedi P., Patil R.D., Kumar M. (2007). Citrinin and endosulfan induced teratogenic effects in Wistar rats. J. Appl. Toxicol..

[15] Singh N.D., Sharma A.K., Dwivedi P., Patil R.D., Kumar M. (2008). Experimentally induced citrinin and endosulfan toxicity in pregnant Wistar rats: Histopathological alterations in liver and kidneys of fetuses. J. Appl. Toxicol..

[16] Singh N.D., Sharma A.K., Dwivedi P., Patil R.D., Kumar M., Ahamad D.B. (2006). Toxicity of endosulfan and citrinin alone and in combination in pregnant rats: Clinico-Haematological and serum biochemical alterations. Ind. J. Vet. Pathol..

[17] Ciegler A., Vesonder R.F., Jackson L.K. (1977). Production and biological activity of patulin and citrinin from *Penicillium expansum*. Appl. Environ. Microbiol..

[18] Vesela D., Vesely D., Jelinek R. (1983). Toxic effects of ochratoxin A and citrinin, alone and in combination, on chicken embryos. Appl. Environ. Microbiol..

[19] Sansing G.A., Lillehoj E.B., Detroy R.W., Miller M.A. (1976). Synergistic toxic effects of citrinin, ochratoxin A and penicillic acid in mice. Toxicology.

[20] Kitchen D.N., Carlton W.W., Hinsman J. (1977). Ochratoxin A and citrinin induced nephrosis in beagle dogs. III. Terminal renal ultrastructural alterations. Vet. Pathol..

[21] Thacker H.L., Carlton W.W. (1977). Citrinin mycotoxicosis in the guinea pig. Food Cosmetics Toxicol..

[22] Siraj M., Phillips T.D., Hayes A.W. (1981). Effects of the mycotoxins citrinin and ochratoxin A on hepatic mixed-function oxidase and adenosine triphosphatase in neonatal rats. J. Toxicol. Environ. Health.

[23] Knasmüller S., Cavin C., Chakraborty A., Darroudi F., Majer B.J., Huber W.W., Ehrlich V.A. (2004). Structurally related mycotoxins ochratoxin A, ochratoxin B, and citrinin differ in their genotoxic activities and in their mode of action in human-derived liver (HepG2) cells: Implications for risk assessment. Nutr. Cancer.

[24] International Agency for Research on Cancer (IARC) (1986). Some Naturally Occurring and Synthetic Food Components, Furocoumarins and Ultraviolet Radiation. IARC Monographs on the Evaluation of Carcinogenic Risks to Humans.

[25] Kanisawa M. (1984). Synergistic effect of citrinin on hepatorenal carcinogenesis of ochratoxin A in mice. Dev. Food Sci..

[26] Jeswal P. (1995). Cumulative effect of ochratoxin A and citrinin on induction of hepatorenal carcinogenesis in mice (*Mus musculus*). Biomed. Lett..

[27] Heussner A.H., Dietrich D.R., O’Brien E. (2006). *In vitro* investigation of individual and combined cytotoxic effects of ochratoxin A and other selected mycotoxins on renal cells. Toxicol. In Vitro.

[28] Grenier B., Oswald I.P. (2011). Mycotoxin co-contamination of food and feed: Meta-Analysis of publications describing toxicological interactions. World Mycotoxin. J..

[29] Pfohl-Leszkowicz A., Molinié A., Tozlovanu M., Manderville R.A., Siantar D.P., Trucksess M.W., Scott P.M., Herman E.M. (2008). Combined Toxic Effects of Ochratoxin A and Citrinin, *in Vitro* and *in Vivo*. Food Contaminats: Mycotoxins & Food Allergens.

[30] Manderville R., Pfohl-Leszkowicz A. (2008). Bioactivation and DNA adduction as a rationale for ochratoxin A carcinogenesis. World Mycotoxin. J..

[31] Trivedi A.B., Doi E., Kitabatake N. (1993). Toxic compounds formed on prolonged heating of citrinin under watery conditions. J. Food Sci..

[32] Trivedi A.B., Hirota M., Doi E., Kitabatake N. (1993). Formation of a new toxic compound, citrinin H1, from citrinin on mild heating in water. J. Chem. Soc..

[33] Kitabatake N., Trivedi A.B., Doi E. (1991). Thermal decomposition and detoxification of citrinin under various moisture conditions. J. Agric. Food Chem..

[34] Van der Merwe K.J., Steyn P.S., Fourie L., Scott D.B., Theron L.L. (1965). Ochratoxin A, a toxic metabolite produced by *Aspergillus ochraceus* Wilh. Nature.

[35] Abarca M.L., Bragulat M.R., Castella G., Cabañes F.J. (1994). Ochratoxin A production by strains of *Aspergillus niger* var. *niger*. Appl. Environ. Microbiol..

[36] Teren J., Varga J., Hamari Z., Rinyu E., Kevei E. (1996). Immunochemical detection of ochratoxin A in black *Aspergillus* strains. Mycopathologia.

[37] Frisvad J.C., Fank J.M., Houbraken J.A.M.P., Kuipers A.F.A., Samson R.A. (2004). New ochratoxin A producing species of *Aspergillus* section *Circumdati*. Stud. Mycol..

[38] Samson R.A., Houbraken J.A.M.P., Kuipers A.F.A., Frank J.M., Frisvad J.C. (2004). New ochratoxin A or sclerotium producing species in *Aspergillus* section *Nigri.*. Stud. Mycol..

[39] Perrone G., Susca A., Epifani F., Mule G. (2006). AFLP characterization of Southern Europe population of *Aspergillus* Section *Nigri* from grapes. Int. J. Food Microbiol..

[40] Larsen T.O., Svendsen A., Smedsgaard J. (2001). Biochemical characterization of ochratoxin A-producing strains of the genus *Penicillium*. Appl. Env. Microb..

[41] Battilani P., Pietri V.A., Giorni P., Formenti S., Bertuzzi T., Toscani T., Virgili R., Kozakiewicz Z. (2007). *Penicillium* populations in dry-cured ham manufacturing plants. J. Food Prot..

[42] Stark A.A. (2005). Threat assessment of mycotoxins as weapons: Molecular mechanisms of acute toxicity. J. Food Prot..

[43] Samson R.A., Frisvad J.C. (2004). *Penicillium* subgenus *Penicillium*: New taxonomic schemes and mycotoxins and other extrolites. Stud. Mycol..

[44] Samson R.A., Pitt J.I. (2000). Integration of Modern Taxonomic Methods for Penicillium. and Aspergillus. Classification.

[45] Pitt J.I., DeVries J.W., Truckseess M.W., Jackson L.S. (2002). Biology and Ecology of Toxigenic *Penicillium* Species. Mycotoxins and Food Safety.

[46] Somma S., Perrone G., Logrieco A.F. (2012). Diversity of black *Aspergilli* and mycotoxin risks in grape, wine and dried vine fruits. Phytopathol. Mediterr..

[47] Rodriguez A., Rodriguez M., Martin A., Nunez F., Cordoba J.J. (2012). Evaluation of hazard of aflatoxin B_1_, ochratoxin A and patulin production in dry-cured ham and early detection of producing moulds by qPCR. Food Contr..

[48] Dall’Asta C., Galaverna G., Bertuzzi T., Moseriti A., Pietri A., Dossena A., Marchelli R. (2010). Occurrence of ochratoxin A in raw ham muscle, salami and dry-cured ham from pigs fed with contaminated diet. Food Chem..

[49] Duarte S.C., Lino C.M., Pena A. (2012). Food safety implications of ochratoxin A in animal-derived food products. Vet. J..

[50] Overy D.P., Frisvad J.C. (2003). New *Penicillium* species associated with bulbs and root vegetables. Syst. Appl. Microbiol..

[51] Blanc P.J., Loret M.O., Goma G. (1995). Production of citrinin by various species of *Monascus*. Biotechnol. Lett..

[52] Samson R.A., Seifert K.A., Kuijpers A.F.A., Houbraken J.A.M.P., Frisvad J.C. (2004). Phylogenetic analysis of *Penicillium* subgenus *Penicillium* using partial β-tubulin sequences. Stud. Mycol..

[53] Hawksworth D.L., Pitt J.I. (1983). A new taxonomy for *Monascus* species based on cultural and microscopical characters. Aust. J. Bot..

[54] EFSA (2006). Opinion of the scientific panel on contaminants in the food chain on request from the commission related to ochratoxin A in food. EFSA J..

[55] Degen G.H., Mayer S., Blaszkewicz M. (2007). Biomonitoring of ochratoxin A in grain workers. Mycotox. Res..

[56] Zimmerli B., Dick R. (1995). Determination of ochratoxin A at the ppt level in human blood, serum, milk and some foodstuffs by high, performance liquid chromatography with enhanced fluorescence detection and immunoaffinity column cleanup: Methodology and Swiss data. J. Chromatogr. B.

[57] Rizzo A., Eskola M., Atroshi F. (2002). Ochratoxin A in cereals, foodstuffs and human plasma. Eur. J. Plant Pathol..

[58] Bonvehi J.S. (2004). Occurrence of ochratoxin A in cocoa products and chocolate. J. Agric. Food Chem..

[59] Molinié A., Faucet V., Castegnaro P., Pfohl-Leszkowicz A. (2005). Analysis of some breakfast cereals on the French market for their contents of ochratoxin A, citrinin and fumonisin B-1: Development of a method for simultaneous extraction of ochratoxin A and citrinin. Food Chem..

[60] Jørgensen K. (2005). Occurrence of ochratoxin A in commodities and processed food—A review of EU occurrence data. Food Addit. Contam..

[61] Clark H.A., Snedeker S.M. (2006). Ochratoxin A: Its cancer risk and potential for exposure. J. Toxicol. Environ. Health Part B.

[62] Napolitano A., Fogliano V., Tafuri A., Ritieni A. (2007). Natural occurrence of ochratoxin A and antioxidant activities of green and roasted coffees and corresponding byproducts. J. Agric. Food Chem..

[63] De Almeida A.P., Alaburda J., Shundo L., Ruvieri V., Navas S.A., Lamardo L.C.A., Sabino M. (2007). Ochratoxin A in Brazilian instant coffee. Braz. J. Microbiol..

[64] Mounjouenpou P., Gueule D., Fontana-Tachon A., Guyot B., Tondje P.R., Guiraud J.P. (2008). Filamentous fungi producing ochratoxin A during cocoa processing in Cameroon. Int. J. Food Microbiol..

[65] Tozlovanu M., Pfohl-Leszkowicz A. (2010). Ochratoxin A in roasted coffee purchased in french super market. Transfer in coffee beverage: Comparison of several methods. Toxins.

[66] Skarkova J., Ostry V., Malir F., Roubal T. (2013). The determination of ultra-trace amounts of ochratoxin A in foodstuffs by HPLC method. Anal. Lett..

[67] Pietri A., Rastelli S., Bertuzzi T. (2010). Ochratoxin A and aflatoxins in liquorice products. Toxins.

[68] Pietri A., Rastelli S., Mulazzi A., Bertuzzi T. (2012). Aflatoxins and ochratoxin A in dried chestnuts and chestnut flour produced in Italy. Food Contr..

[69] Bertuzzi T., Gualla A., Morlacchini M., Pietri A. (2013). Direct and indirect contamination with ochratoxin A of ripened pork products. Food Control.

[70] Schmidt-Heydt M., Graf E., Batzler J., Geisen R. (2013). The application of transcriptomics to understand the ecological reasons of ochratoxin A biosynthesis by *Penicillium nordicum* on sodium chloride rich dry cured food. Trends Food Sci. Tech..

[71] Biancardi A., Piro R., Galaverna G., Dall’Asta C. (2013). A simple and reliable liquid chromatography-tandem mass spectrometry method for determination of ochratoxin A in hard cheese. Inter. J. Food Sci. Nutr..

[72] Dall’Asta C., De Dea Lindner J., Galaverna G., Dossena A., Neviani E., Marchelli R. (2008). The occurrence of ochratoxin A in blue cheese. Food Chem..

[73] Pfohl-Leszkowicz A., Petkova-Bocharova T., Chernozemsky I.N., Castegnaro M. (2002). Balkan endemic nephropathy and the associated urinary tract tumours: Review on etiological causes, potential role of mycotoxins. Food Addit. Contam..

[74] Castegnaro M., Canadas D., Vrabcheva T., Petkova-Bocharova T., Chernozemsky I.N., Pfohl-Leszkowicz A. (2006). Balkan endemic nephropathy: Role of ochratoxins A through biomarkers. Mol. Nutr. Food Res..

[75] Markov K., Pleadin J., Bevardi M., Vahčić N., Sokolić-Mihalak D., Frece J. (2013). Natural occurrence of aflatoxin B_1_, ochratoxin A and citrinin in Croatian fermented meat products. Food Contr..

[76] Reddy R.V., Berndt W.O., Sharma R.P., Salunk D.K. (1991). Citrinin. Mycotoxins and Phytoalexins.

[77] Jimenez M., Mateo R., Querol A., Huerta T., Hernandez E. (1991). Mycotoxins and mycotoxigenic moulds in nuts and sunflower seeds for human consumption. Mycopathologia.

[78] Dietrich R., Schmid A., Märtlbauer E. (2001). Citrinin in fruit juices. Mycotox. Res..

[79] Meister U. (2004). New method of citrinin determination by HPLC after polyamide column clean-up. Eur. Food Res. Technol..

[80] El Adlouni C., Tozlovanu M., Naman F., Faid M., Pfohl-Leszkowicz A. (2006). Preliminary data on the presence of mycotoxins (ochratoxin A, citrinin and aflatoxin B_1_) in black table olives “Greek style” of Moroccan origin. Mol. Nutr. Food Res..

[81] Heperkan D., Meric B.E., Sismanoglu G., Dalkiliç G., Güler F.K. (2006). Mycobiota, mycotoxigenic fungi, and citrinin production in black olives. Adv. Exp. Med. Biol..

[82] Nguyen Minh T., Tozlovanu M., Tran Thi L., Pfohl-Leszkowicz A. (2007). Occurrence of aflatoxin B1, citrinin and ochratoxin A in rice in five provinces of central region in Vietnam. Food Chem..

[83] Bailly J.D., Querin A., le Bars-Bailly S., Benard G., Guerre P. (2002). Citrinin production and stability in cheese. J. Food Prot..

[84] EFSA (2012). Scientific Opinion on the risks for public and animal health related to the presence of citrinin in food and feed. EFSA J..

[85] Kumari H.P.M., Naidu K.A., Vishwanatha S., Narasimhamurthy K., Vijayalakshmi G. (2009). Safety evaluation of *Monascus purpureus* red mould rice in albino rats. Food Chem. Toxicol..

[86] Zheng Y., Xin Y., Guo Y. (2009). Study on the fingerprint profile of *Monascus* products with HPLC-FD, PAD and MS. Food Chem..

[87] Gordon R.Y., Cooperman T., Obermeyer W., Becker D.J. (2010). Marked variability of monacolin levels in commercial red yeast rice products: buyer beware!. Arch. Inter. Med..

[88] Samsudin N.I., Abdullah N. (2013). A preliminary survey on the occurrence of mycotoxigenic fungi and mycotoxins contaminating red rice at consumer level in Selangor, Malaysia. Mycotoxin Res..

[89] Wu C.L., Kuo Y.H., Lee C.L., Hsu Y.W., Pan T.M. (2011). Synchronous high-performance liquid chromatography with a photodiode array detector and mass spectrometry for the determination of citrinin, monascin, ankaflavin, and the lactone and acid forms of monacolin K in red mold rice. J. AOAC Int..

[90] Dimmer T. (2013). personal communication.

